# Understanding Chinese EFL learners’ anxiety in second language writing for the sustainable development of writing skills

**DOI:** 10.3389/fpsyg.2022.1010010

**Published:** 2022-11-04

**Authors:** Yue Yu, Dandan Zhou

**Affiliations:** ^1^College of International Students, Southeast University, Nanjing, China; ^2^School of Foreign Studies, Nanjing University, Nanjing, China

**Keywords:** second language writing anxiety, Chinese EFL learners, cultural influences, ethnic culture, local culture, academic culture, disciplinary culture

## Abstract

To add to the currently limited research on the degree of cultural uniqueness of Chinese EFL learners’ anxiety and the multidimensional nature of second language writing anxiety (SLWA), the present qualitative study used think-aloud protocol and interview to examine Chinese EFL learners’ three dimensions of SLWA and the related variables, so as to probe into this problem that could pose an obstacle to sustainable second language writing. Findings showed that Chinese EFL learners experienced much Cognitive Anxiety, but relatively little Avoidance Behavior. Learner-internal, teacher-related, and human-external variables interacted with SLWA in a dynamic way. To a certain degree, Chinese EFL learners showcased uniqueness in some aspects of SLWA, under cultural influences, regarding ethnic culture, local culture, academic culture, and disciplinary culture.

## Introduction

Anxiety has long been established as a common factor that affects the sustainable development of students’ learning. It is a prevalent phenomenon, as opposed to being experienced in isolated cases (Trang et al., 2013). Further, anxiety often arises in different stages of students’ learning (Onwuegbuzie et al., 2000). As an affective factor that continues to attract researchers’ attention, language anxiety (LA) has become one of the most frequently studied emotions in the field of SLA (Horwitz, 2010; Dewaele, 2012; MacIntyre, 2017; MacIntyre and Vincze, 2017; Jiang and Dewaele, 2019). Research on anxiety usually involves the effects of anxiety, the (mutual) relationship between anxiety and performance, and the internal or social nature of anxiety. Considering these issues, recent research on anxiety has demonstrated significant differences in levels of foreign language anxiety (FLA) between Asian learners and the others (Dewaele and MacIntyre, 2014; MacIntyre et al., 2019). Particularly, more than one third of Chinese college students encounter foreign language anxiety in English class (Liu and Jackson, 2008; Shao et al., 2013), and some studies emphasized the uniqueness of Chinese EFL learners’ FLA as well as highlighted the importance to investigate how cultural factors could shape the interactions between learner-internal and learner-external variables on anxiety (MacIntyre and Vincze, 2017; Jiang and Dewaele, 2019).

Academic writing is often regarded as an “emotionally laden process,” particularly for second language writers (Han and Hyland, 2019, p. 1), and second language writing anxiety (SLWA) in this process could not be ignored, since it interferes with the sustainable development of second language writing. Identified as a skill-specific anxiety, SLWA should be measured differently from general anxiety, foreign language classroom anxiety (FLCA), and first language writing anxiety (MacIntyre and Gardner, 1989; Cheng et al., 1999; Cheng, 2008). Since SLWA might be related to but is different from the general FLA, conclusive remarks could not be made as for whether Chinese EFL learners also experience SLWA differently from other learners in the rest of the world, which deserves further research. Some researchers have realized the complex and multidimensional nature of anxiety and focused on the three dimensions of SLWA identified by Cheng (2004), based on Lang’s (1971) tripartite model which conceptualizes anxiety as comprising physiology, behavior, and cognition. Accordingly, Cheng’s (2004) model comprises Somatic Anxiety, Avoidance Behavior, and Cognitive Anxiety. Some studies explored these three dimension of SLWA, and these studies indicated that EFL learners in China usually experienced Cognitive Anxiety the most (Chiang, 2012; Sun and Fan, 2022; Zhang and Zhang, 2022), while EFL learners outside China usually experienced Avoidance Behavior the most (Xiao and Wong, 2014; Salem, 2022). Although these studies have provided more information than research using unidimensional measurement (Cheng, 2017), they generally possessed a quantitative nature, lacking qualitative and in-depth investigation.

The present study combines these two lines of research, adopting a qualitative design to pursue a deeper understanding of how Chinese EFL learners experience Somatic Anxiety, Avoidance Behavior, and Cognitive Anxiety during an English writing task, and also explores to what extent each dimension of SLWA of Chinese EFL learners is culturally unique. Students’ performance and related variables were also analyzed from the perspective of four dimensions of culture, namely ethnic, local, academic, and disciplinary culture (Flowerdew and Miller, 1995), trying to examine the respective influence of each dimension of culture on each dimension of SLWA.

## Literature review

### Trends of research on anxiety

Just as MacIntyre (2017, p. 27) has summarized, previous research on anxiety mainly concerned three issues or trends: (1) whether anxiety is facilitating or debilitating, (2) whether anxiety is “a cause or an effect of language performance,” and (3) whether anxiety is “an internal state or socially constructed.”

As for the effects of anxiety, there has been considerable debate. Many researchers identified the negative effects of anxiety. MacIntyre and Gardner’s (1991) review suggested that anxiety generally impairs language learning and production. Doughty and Long (2005, p. 147) also regarded anxiety as debilitating, calling for an “anxiety-free social interaction,” which will raise levels of L2 proficiency. On the other hand, some experts pointed out that anxiety also has positive effects on learning, believing that facilitating and debilitating anxiety are beneficial and detrimental, respectively (Scovel, 1978; Trang et al., 2013). According to some scholars, the relationship between anxiety and performance is not a “simple linear one” (Ellis, 1994, p. 482), but a “curvilinear” one (Gass et al., 2008, p. 400), suggesting that low levels of anxiety are helpful, while high levels of anxiety are harmful (Krashen, 1981). All these showcase the “schizophrenic nature” of anxiety (Larsen-Freeman and Long, 1991, p. 326). However, MacIntyre’s (2017) review conceptualized anxiety as only debilitating, negating all the possibilities of facilitating anxiety.

As for the relationship between anxiety and language performance, earliest studies generally focused on the effects of anxiety, until Sparks and Ganschow (1995) held that anxiety resulted from the lack of language aptitude and the weakness in linguistic coding. Xiao and Wong’s (2014, p. 590) review recognized a “potential negative association” between FLA and academic achievement, and the intensification of a vicious cycle in this process, indicating that anxiety and language performance exert mutual effects on each other. MacIntyre’s (2017) review also regarded anxiety as both a cause and consequence of language performance.

As for whether anxiety is internally decided or socially dynamic, it was originally unclear whether it should be “a matter of personality, an emotional reaction to a situation, or a combination” (Gass et al., 2008, p. 400). Horwitz et al. (1986) summarized the three dimensions of issues associated with FLA, namely (1) communication apprehension, specifically situational communication apprehension; (2) test anxiety; and (3) a fear of negative evaluation. These three dimensions seemed to encompass both the internal trait and the emotional reaction to social conditions. After years of research, MacIntyre (2017) proposed that language anxiety “is influenced by internal physiological processes, cognitive and emotional states along with the demands of the situation and the presence of other people, among other things, considered over different timescales. Anxiety has both internal and social dimensions” (p. 28). This indicates the importance to take both learner-internal characteristics and language learning environment into account with respect to the discussion on anxiety. Besides, the emerging dynamic approach proposes the continuous interaction between anxiety and other factors, which could fluctuate over time. Difficulty in communication apprehension, frustration over the frequent tests, and fear of negative evaluation were originally regarded as useful building blocks to conceptualize LA in order to further explore and understand its nature and origins (Horwitz et al., 1986), which gained support from later studies and were supplemented with other factors like difficulty of the courses, unfamiliarity with the foreign culture, worry about comprehension, high social expectation, peer pressure, ineffective learning strategies, low language learning interest and motivation, low language proficiency level, negative attitudes towards the target language, negative attitudes towards the teacher (Saito and Samimy, 1996; Yan and Horwitz, 2008; Zhao et al., 2013; De Costa, 2015; Jiang and Dewaele, 2019), to name a few. Most of these factors are not fixed, but could change at any time, and then lead to the changes of anxiety. Those internally stable factors like gender and language aptitude also exist, but were regarded as remote sources of anxiety (Yan and Horwitz, 2008).

Among the three trends mentioned above, recent research attaches great attention to the third trend, and considers the identity of students experiencing anxiety, especially Chinese EFL learners. MacIntyre et al. (2019) conducted a quantitative research into the emotional underpinnings of Gardner’s Attitudes and Motivation Test Battery, and the results showed significant differences between 158 Chinese foreign language students and 303 foreign language learners outside China. Particularly, Chinese participants reported significantly higher values on Foreign Language Use Anxiety and FLCA. Their study involved the third issue among the three trends mentioned above, namely the social dynamic construction of anxiety. Specifically, they illustrated variables and factors related to anxiety. They emphasized the impact of specific educational and sociocultural factors on anxiety. The differences were thus speculatively attributed to the strong exam-oriented nature of Chinese education, as well as the lack of opportunities to communicate with members of the target culture.

The mixed-method study of Jiang and Dewaele (2019) investigated the foreign language enjoyment (FLE) and FLCA of 564 Chinese EFL learners in the context of English Listening and Speaking classes, and compared the results with the international sample adopted in Dewaele and MacIntyre (2014) study. Similar to the abovementioned study by MacIntyre et al. (2019), the study by Jiang and Dewaele (2019) also involved the third issue among the three trends mentioned above, namely the social dynamic construction of anxiety. They elaborated on variables and factors related to anxiety, including learner-internal and teacher-related variables. Results showed that Chinese students experienced more FLA, which could be attributed to Chinese educational context that traditionally focuses on written English and is associated with exam-oriented culture. However, the independent variables predicting Chinese EFL learners’ FLCA were not fundamentally unique compared with learners outside China, since they all revealed that FLCA was mostly predicted by learner-internal variables, rather than by teacher-related variables.

As such, taking into account the third trend in the field of anxiety, previous research has reported certain unique features of Chinese EFL learners’ general anxiety and/or FLCA. To follow this line of research, the present study concentrates on Chinese EFL learners’ SLWA so as to explore whether this uniqueness is able to be generalized to skill-specific anxiety, specifically SLWA.

### Second language writing anxiety

Skill-specific anxiety, such as SLWA, is distinguished from general anxiety, FLCA, and first language writing anxiety, especially in measurement (MacIntyre and Gardner, 1989; Cheng et al., 1999; Cheng, 2008). Compared with research on second language listening, speaking, and reading anxiety, less attention was paid to SLWA in early days (Woodrow, 2011). Recent years have witnessed a growth of studies on SLWA, but still, more studies are needed, and only a few studies measured SLWA through a multidimensional lens, according to Cheng’s (2017) review. Based on Lang’s (1971) tripartite model of anxiety, Cheng (2004) proposed the framework of three dimensions of anxiety, namely Somatic Anxiety, Avoidance Behavior, and Cognitive Anxiety.

Somatic Anxiety is related to “increased physiological arousal” (Cheng, 2004, p. 325). It is often reflected in people’s physical feelings and behavior, like “upset stomach, pounding heart, excessive sweating, and numbness” (Cheng, 2004, p. 318). Most of the symptoms that showcase uneasiness during L2 writing are categorized as the revelations of Somatic Anxiety.

Avoidance Behavior indicates a tendency of avoidance to writing, It concerns people’s habits to avoid issues that involve second language writing, such as “procrastination, withdrawal, and avoidance” (Cheng, 2004, p. 318). It should be noted that Avoidance Behavior is not equal to the exact behavior. Instead, it is a dimension of anxiety, which indicates a tendency to perform certain behavior.

Cognitive Anxiety is associated with worry or fear of negative evaluation. It suggests that people are afraid of losing face or being looked down upon, reflected in various aspects like “worry, preoccupation, and negative expectations” (Cheng, 2004, p. 318). It is not equal to the exact negative comments, but a dimension of anxiety associated with the fear for negative comments.

For the measurement of the three dimensions of anxiety, Cheng (2004) designed the Second Language Writing Anxiety Inventory (SLWAI), using three subscales to measure Somatic Anxiety, Avoidance Behavior, and Cognitive Anxiety separately There are 22 items in total, with 7 items on the Somatic Anxiety subscale, 7 items on the Avoidance Behavior subscale, and 8 items on the Cognitive Anxiety subscale (Cheng, 2004, p. 326). For instance, items on the Somatic Anxiety subscale ask students whether they tremble or perspire, items on the Avoidance Behavior subscale ask students whether they try to avoid writing English essays, items on the Cognitive Anxiety subscale ask students whether they worry about receiving low scores (Cheng, 2004, p. 324). According to Cheng (2004, p. 319), the 5-point Likert-scale is adopted, from 1 (this statement is never or almost never true of me) to 5 (this statement is completely or almost completely true of me), and a higher score indicates a higher level of SLWA. The mean of 3.5–5.0 suggests a high degree of anxiety; 2.5–3.4 suggests a medium degree of anxiety; 1.0–2.4 suggests a low degree of anxiety (Oxford and Burry-Stock, 1995, p. 12).

Several researchers employed Cheng’s (2004) SLWAI in their studies. Chiang (2012) investigated 156 Chinese high school female students’ multidimensional perfectionism and SLWA. Results showed that participants experienced Cognitive Anxiety the most (3.17 on a 5-point Likert scale), followed by Avoidance Behavior (3.02), and Somatic Anxiety the least (2.91), and that there were significant differences between the level of Cognitive Anxiety and that of the other two dimensions of anxiety. Overall perfectionism had a small but significant positive relationship with the overall SLWA (*r* = 0.176, *p* < 0.05) as well as Somatic Anxiety (*r* = 0.201, *p* < 0.05) and Cognitive Anxiety (*r* = 0.238, *p* < 0.01).

Xiao and Wong (2014) examined the four language-skill based anxiety of 87 Chinese heritage language learners enrolled in separated heritage-track courses at two American universities. For the measurement of writing anxiety, they employed Cheng’s (2004) SLWAI. The results showed that they experienced Avoidance Behavior the most (3.30), followed by Cognitive Anxiety (2.97), and Somatic Anxiety the least (2.57).

Tsiriotakis et al. (2017) investigated 177 participants’ anxiety with Cheng’s (2004) SLWAI. The participants were from two mainstream primary schools in the city of Chania, Crete, and were all of Greek background. Participants were divided into experimental group and control group, the former received the Self-Regulated Strategy Development (SRSD) writing instruction, while the latter did not. Results suggested the negative effects of anxiety on writing production, language achievement, and language learning.

Zarrinabadi and Rezazadeh (2020) selected 210 Iranian female EFL learners, who were Persian native speakers, and investigated the influences of feedback, feed up (comments on goals and students’ success in achieving goals, e.g., “You have done your task successfully. This enumeration paragraph is good.”) and feed forward (comments on the next step in learning during the semester, e.g., “In the next session, we will learn how to write supporting sentences.”) on writing motivation, writing self-efficacy, and writing anxiety. For the measurement of writing anxiety, they selected Cheng’s (2004) SLWAI. Their results showed that participants’ writing anxiety could be significantly lowered by giving feed forward, as it could give the participants the feeling that they successfully accomplished the current step and thus could go to the next one.

Tahmouresi and Papi (2021) selected 85 EFL university students in Iran, and explored how their L2 writing achievement was predicted by motivation and emotions, the latter of which included enjoyment and anxiety. To measure anxiety, they employed Cheng’s (2004) SLWAI. Their findings suggested that L2 writing anxiety negatively predicted L2 writing achievement.

Heidarzadi et al. (2022) explored the roles of epistemic beliefs (EBs) and writing self-efficacy (WSE) in predicting SLWA among 240 Iranian EFL learners. Results showed that EBs directly and significantly influenced their SLWA. Besides, students who had a higher level of EBs experienced more SLWA, and those who had a higher level of WSE experienced less SLWA.

Salem (2022) examined the impact of learning in a sheltered Internet environment on skills and writing anxiety. For the measurement of anxiety, they employed Cheng’s (2004) SLWAI. Participants were undergraduates and graduates in Egypt, divided into three groups, namely groups of sheltered online instruction (the experimental group, 15 students), unsheltered online instruction (free Google search) (the first control group, 19 students), and sheltered offline instruction (the second control group, 20 students). According to the findings, a commonality among the three groups was that all of them experienced Avoidance Behavior the most, no matter in pre-test or in post-test.

Sun and Fan (2022) investigated 73 Chinese business English majors’ writing performance and writing anxiety, divided into the experimental group (39 participants) that received the AWE (Automated Writing Evaluation)-aided assessment, and the control group (34 participants) that obtained an instructor-only assessment. They used Cheng’s (2004) SLWAI to measure anxiety. The results suggested that for both groups, participants experienced Cognitive Anxiety the most, no matter in pre-test or in post-test. Besides, writing anxiety had no significant mediation effect on the relationship between the assessment approach and writing performance.

Zhang and Zhang (2022) selected 55 English majors in China and investigated the impact of procrastination on SLWA, considering the mediating role of SLWA. Results showed that participants experienced Cognitive Anxiety the most (3.027), followed by Avoidance Behavior (2.906), and Somatic Anxiety the least (2.621). Besides, academic procrastination positively influenced all the three dimensions of SLWA significantly. SLWA played a mediating role in the impact of procrastination on L2 writing.

All in all, these studies of SLWA indicated that EFL learners in China usually experienced Cognitive Anxiety the most (Chiang, 2012; Sun and Fan, 2022; Zhang and Zhang, 2022), while EFL learners outside China usually experienced Avoidance Behavior the most (Xiao and Wong, 2014; Salem, 2022). No research on SLWA has attended to these differences, as their research focus was usually on the multi-dimensional nature of anxiety, or other variables related to anxiety. As has been mentioned, Chinese educational system is different from those in other countries, reflected in various features such as the exam-oriented nature (Jiang and Dewaele, 2019; MacIntyre et al., 2019). It might be thus meaningful to explore whether the different features of the Chinese educational system could contribute to the differences in SLWA between Chinese EFL learners and others outside China.

As such, “Second language writing anxiety” has introduced another line of research apart from the line of research reviewed in “Trends of research on anxiety”. To follow this line of research mentioned in “Second language writing anxiety,” the present study also attends to the multidimensional nature of anxiety, and tries to explore the Somatic Anxiety, Avoidance Behavior, and Cognitive Anxiety of Chinese EFL learners during the completion of an English writing task.

### Need for qualitative approach

Although previous studies of the three dimensions of SLWA displayed a more complicated picture of SLWA than those from a one-dimensional perspective (Cheng, 2017), they usually relied on quantitative research and self-report data by employing SLWAI. Thus, those authors might find it uneasy to explain certain phenomena, such as the reasons for the differences between Chinese EFL learners and those students outside China regarding their experience of different dimensions of anxiety. These do call for qualitative research as a supplement, in order to further explore the three dimensions of anxiety in writing process.

Previous research on anxiety could mirror the contribution of qualitative approach. Aichhorn and Puck (2017) adopted an inductive case study research design, and they conducted semi-structured open-ended interviews on 22 informants from a variety of countries, including Austria, Slovenia, Finland, Sweden, etc. Their research involved the first issue among the abovementioned three trends, namely effects of anxiety. Their findings indicated that anxiety was reflected in communication avoidance and withdrawal, as well as code-switching, exerting a considerable impact on interpersonal communication, disrupting sharing of content and impairing social relations.

Dryden et al. (2021) conducted a qualitative study, adopting the method of Linguistic Ethnography (LE), specifically semi-structured interviews and focus group discussions (FGD), so as to explore how four migrant adult EFL learners in Australia experienced FLA. Their study involved the first and third issues among the abovementioned three trends on research, namely the effects of anxiety and the social dynamic construction of anxiety. As for the effects of anxiety, their findings presented the negative effects of FLA on their emotions and physical states, such as forgetfulness, temporal loss of the ability to interact with others, crying, and the desire to avoid speaking English. As for the social dynamic construction of anxiety, their findings suggested that anxiety resulted from their interaction with members of the host society, and that a translanguaging environment could largely alleviate their anxiety.

Yu et al. (2021) conducted a qualitative study, collecting data from semi-structured interviews, sample teacher feedback and student writing, and teaching materials, in order to explore the negative emotions (including anxiety) of 27 EFL writing teachers in Chinese universities. Their study involved the first and third issues among the abovementioned three trends on research, namely the effects of anxiety and the social dynamic construction of anxiety. As for the effects of anxiety, their findings suggested that anxiety negatively affected the feedback practice. As for the social dynamic construction of anxiety, the results showed that participants were experiencing anxiety resulting from high workload. Two teachers also mentioned that their anxiety resulted from the contradiction between their beliefs and the institutional criteria rather than the institutional requirements themselves.

To obtain deeper and richer findings of the cultural uniqueness of anxiety experienced by Chinese EFL learners, the qualitative approach can be of great assistance. In the two studies mentioned in “Trends of research on anxiety,” MacIntyre et al. (2019) employed a quantitative approach, while Jiang and Dewaele (2019) employed a mixed-method approach. Both of these studies yielded abundant harvests, but when it comes to the variables and factors that might explain or affect the uniqueness of Chinese EFL learners’ anxiety, Jiang and Dewaele (2019) were able to find more learner-internal and teacher-related variables thanks to the qualitative analysis of participants’ answers to the two open-ended questions.

As such, no research so far has investigated the cultural uniqueness of Chinese EFL learners’ SLWA from a qualitative perspective, and this deserves further research. Through the qualitative lens, especially think-aloud protocol, attention could be paid to the exact process of second language writing, in order to capture the uniqueness by closely examining the social dynamic construction of anxiety. These can help illustrate how cultural factors are related to such uniqueness by shaping the interactions between learner-internal and learner-external variables on anxiety (MacIntyre and Vincze, 2017; Jiang and Dewaele, 2019). The present study adopts a qualitative approach, and tries to explore the Somatic Anxiety, Avoidance Behavior, and Cognitive Anxiety by Chinese EFL learners through the completion of an English writing task. The analysis was based on cultural perspectives. It should be noted that the issue of cultural factors can be thorny and complex, since it is difficult to “tease out culture from language and other background factors” (Pecorari, 2015, p. 96). Fortunately, Flowerdew (2015) argued that the aforementioned problem could be alleviated by exploring the four dimensions of culture, including ethnic, local, academic, and disciplinary culture (Flowerdew and Miller, 1995). Therefore, the analysis of their uniqueness under the influence of cultural factors would be based on the framework of Flowerdew and Miller’s (1995) four dimensions of culture, namely ethnic, local, academic, and disciplinary culture. This framework shall be applicable to interpretation in this study, since it encompasses various aspects of students’ cultural background in a broad sense, thereby providing a systematic construct for the factors that might influence students’ SLWA. So far, hardly could any other framework provide such a rich notion of culture.

Ethnic culture concerns the “culturally based, social–psychological features” (Flowerdew and Miller, 1995, p. 346). It involves the social-psychological aspect, associated with traditional ideology. It is often associated with a person’s thinking pattern and habits shaped by the conventions of an ethnic group.

Local culture indicates features of the local setting that are familiar to members of a certain society. It concerns the local features in people’s life. It is also related to people’s personal background concerning the social environment or context they are in.

Academic culture concerns the features requiring “an understanding of the particular academic values, assumptions, roles, and so on of a given society” (Flowerdew and Miller, 1995, p. 346). It mirrors a person’s thinking and practice in the academic field and in the academic institutions.

Disciplinary culture concerns the issues exclusive to a discipline. It reflects certain theories and terms of a particular academic discipline. It is also associated with principles and practices typical of teachers and students of different disciplines. It encompasses different writing patterns, different learning procedures, and the different focus during writing in different disciplines.

## Research questions

The present study adopts a qualitative approach, and tries to explore the Somatic Anxiety, Avoidance Behavior, and Cognitive Anxiety by Chinese EFL learners through the completion of an English writing task. The analysis was based on cultural perspectives. Specific research questions were:

1. How do Chinese EFL learners experience SLWA, specifically Somatic Anxiety, Avoidance Behavior, and Cognitive Anxiety?
2. What factors are related to SLWA according to the behavior and opinions of the participants?

## Methodology

### Participants

Participants were sampled based on their relationships with the researchers and attitudes towards the think-aloud protocol. They should neither be unknown or too close to the researchers, so that their performance would be less likely to be affected by the researchers’ presence during the think-aloud session. Besides, they should not hold negative attitudes towards the think-aloud protocol, in order to ensure that their anxiety would be mainly related to SLWA. Participants were recruited at the end of an academic year by means of the researchers’ invitation. All of them were in the same university as the researchers, but did not attend any class taught by the researchers or had frequent interactions with the researchers. In other words, the researchers and them knew each other, but were not so close. Besides, they all assured that they were willing to take part in a think-aloud session. They also declared that they would not be affected by the researcher’s presence during their writing, which was confirmed again by themselves after the think-aloud session.

The six participants, Alice, Bella, Carol, Doris, Emily, and Fiona (all names are pseudonyms), were all female undergraduate Chinese EFL learners from one university in China. They were all 21-year-old junior English majors, coming from three different classes. They had passed TEM-4, and had adequate academic writing experiences, including essay-writing assignments and in-class writing activities. They also answered essay questions regularly and openly on a website to meet course requirements. They had to use English when finishing essay-writing assignments and participating in in-class writing activities, but free to answer on-line questions in either Chinese or English. Among the six participants, two of them (Alice and Emily) habitually answered on-line essay questions in Chinese, while the other four (Bella, Carol, Doris, and Fiona) in English.

### Data collection

In such a qualitative study, the roles of the researchers as the “primary data collection instrument” require the “identification of personal values, assumptions, and biases at the outset of the study” (Creswell and Creswell, 2018, p. 281). As the researchers were from the same university and same major as the participants, it was relatively easy for the researchers to understand participants’ behavior and explanations due to many similarities in experiences. The researchers have certain experiences in anxiety research and cultural research, helping to relate participants’ behavior to anxiety or cultural influences. However, the researchers might also bring certain biases to this study and thus impairing validity to some extent. Although much effort has been made to ensure the objectivity, these biases might still affect the data analysis procedure, specifically the way to interpret the data. Although the participants were not that close to the researchers, the researchers might still analyze their behavior and thoughts based on their personal characteristics, rather than only based on their performance in the present study. This issue was addressed by the reflexivity and negative case sampling, as the researchers frequently reflected on their own biases and potential tendencies in a critical way, and probed into the cases that did not confirm their expectations and explanations.

The six participants met one of the researchers individually. Prior to data collection, all participants received a Participant Information Sheet (brief procedure of the study, ensurance of the confidentiality of their data, and researchers’ contact information). They also filled a consent form. Participants were not informed of the detailed purposes of the study until the second half of the interviews, when they had completed the think-aloud sessions and explanations of the behavior in think-aloud sessions. In this way, they did not know that the study focused on anxiety when taking part in think-aloud sessions, so that they could perform naturally, instead of consciously avoiding anxiety during the think-aloud sessions.

#### Think-aloud protocol

The participants first took part in the think-aloud sessions. Validity of the think-aloud protocol concerns reactivity and veridicality (Zhang and Zhang, 2020), both of which was ensured in the present study. As for reactivity, much research has suggested that the degree of severity of reactivity effects is rather minor (Zhang and Zhang, 2020), which was assured by all the participants after the training session as well as the formal session of think-aloud. As for veridicality, following Zhang and Zhang’s (2020) suggestions, clear instruction and training were provided, so as to help obtain high-quality and valid data.

With their consent, the researcher gave participants a brief instruction on think-aloud session, demonstrating how to describe their mental processes. Participants were required to verbalize everything in their mind during writing process. Then, all the participants completed a practice task, writing a 100-word argumentative paragraph to discuss whether university students should do part-time job. The focus of this practice and training aimed to help participants become used to this method and to help them find their difficulties in the think-aloud sessions. This practice session was audio-recorded, and then the researcher listened to the recordings together with the participants and had discussions with them in order to help them resolve difficulties they encountered.

Then, in the formal think-aloud sessions, three of the participants (Alice, Bella, and Carol) were required to finish a 300-word argumentative essay on the topic of “whether daily homework is necessary for students” within 30 min. The other three (Doris, Emily, and Fiona) were required to finish a 300-word essay on the same topic, but with no time limit. The time limit helped to create two common conditions of writing. The former condition represented in-class writing activities, in which 30 min was the usual time limit assigned by teachers, while the latter condition represented after-class writing activities, in which no time limit was assigned. The 30 min included the time both for writing and speaking out their thoughts. Participants claimed after their practice sessions that verbalizing their thoughts did not largely disturb their writing process, especially when they gradually became familiar with this method towards the end of the practice sessions. Participants were informed that their essays would be scored. The researcher audio-recorded their thoughts, observed their behavior and took notes in the whole process. The focus of the behavior observation was their facial expressions, their trivial body movements, and the smoothness of their voice.

#### Interview

Interviews were conducted immediately after the think-aloud sessions, in Chinese (participants and the researcher’s mother tongue), in a face-to-face manner.

The first part was a non-structured interview. The researcher communicated with participants and asked them to explain some of their behavior during the think-aloud session, without revealing the topic of anxiety, so as to enable them to explain their behavior in a natural way, without purposefully relating to or avoiding anxiety. The researcher also asked about their writing attitudes and habits based on their previous behavior and utterances.

The second half of the interview was a semi-structured interview. The researcher informed the participants of the detailed purposes of the study and asked about their past experiences, attitudes, and strategies related to anxiety. They were directly asked about their self-perceived sources and influences of anxiety. They also reported the strategies which their teachers and they had employed to deal with SLWA. The questions of the interview were developed based on the discussion of the two researchers and validated by conducting a pilot interview. Revisions were made to the interview questions based on the results of the pilot interview after further discussion of the researchers. In this way, reliability and validity of the interview could be better ensured.

The whole process was audio-recorded. There was no strict time limit for the interviews, and each participant’s interview lasted for 30 to 40 min.

### Data analysis

Qualitative data were transcribed and combined with observation notes. The transcriptions of the think-aloud data totalled 10,909 words (including Chinese characters and English words), while those of the interview data totalled 10,490 words (mainly Chinese characters). All the examples listed in this article were translated into English.

Then the transcribed data went through the procedures of coding, completed by two coders, and all the discrepancies were discussed until agreement was reached. Also, the invited coder did not know the six participants, so that she would find it easier to maintain an objective manner during coding. The study followed a grounded theory coding approach as proposed by Corbin and Strauss (2008), which contains three major steps, namely open coding, axial coding and selective coding.

During the open coding, the transcripts were read in great detail. The coding adopted certain words to mark the symptoms and variables related to anxiety. A few examples are given as follow. The sentence “I do not know what to write now” was captured under the code “minds going blank.” The sentence “That’s enough, I do not want to continue my writing” was captured under the code “reluctance to write.” The sentence “I do not want to get a low score” was captured under the code “fear of low scores.” The sentence “My grammatical knowledge is so poor” was captured under the code “low self-esteem.” The sentence “My writing teacher often pointed out many mistakes in my assignment” was captured under the code “teacher’s strictness.” The sentence “Oh gosh the time is running out” was captured under the code “time constraint.” The commonalities among different codes were also investigated, providing the foundation for classifying them into different categories.

During the axial coding, we reassembled the categories formed during the open coding, and tried to connect them with issues underlying them. Some categories were classified into different revelations of Somatic Anxiety, Avoidance Behavior, and Cognitive Anxiety, while others were classified into different variables related to anxiety, namely learner-internal, teacher-related, and human-external variables. For instance, codes like “minds going blank” and “heart beating fast” were classified into the category “revelations of Somatic Anxiety,” while codes like “perfectionism” and “low self-esteem” were classified into the category “learner-internal variables.” The axis in these cases should be revelations of anxiety or variables related to anxiety.

In the selective coding, the main theme emerged, which should be “three dimensions of SLWA shaped by three types of variables.” This theme was related to all the categories.

The coding was accompanied by memoing (memo keeping). During the memoing process, we kept down all the phenomena that might be related to cultural features, such as the collectivist approach reflected in students’ writing habits. We stopped data analysis when we had reached theoretical saturation and new data analysis did not elicit any new or significant insights.

Then, all the revelations of anxiety or variables related to anxiety were compared with those in previous research. The differences were analyzed from cultural perspectives, specifically ethnic, local, academic, and disciplinary culture. Data analysis in this stage focused on how different dimensions of cultural factors could influence the revelations of the three dimensions of anxiety as well as the three types of variables related to anxiety, thereby shaping the cultural uniqueness of Chinese EFL learners’ SLWA. The basis for such analysis had three sources. First, participants’ behavior and thoughts mirroring cultural features, which were kept in the memo. Second, previous literature concerning cultural issues. Third, the researchers’ interpretation based on past experiences.

To maintain the trustworthiness of the study, this study followed the set of conventional criteria, including internal validity, external validity, reliability, and objectivity (Lincoln and Guba, 1985). For the present study, internal validity was mainly ensured by the methods triangulation, including think-aloud protocol and interview. External validity was mainly ensured by describing the context as well as data collection and analysis in detail, and comparing findings of the present study with those of the previous ones. Reliability was mainly ensured by the inclusion of an invited coder and the discussion on all the discrepancies until agreement was reached. Objectivity was mainly ensured by giving participants adequate training and temporarily concealing the topic of anxiety so as to help them complete the think-aloud session in a natural manner.

## Results

### Anxiety in L2 writing process

Table 1 summarizes participants’ experiences of SLWA according to their behavior in the think-aloud session and their reports in the interview.

**Table 1:** Participants’ experiences of second language writing anxiety (SLWA).

Anxiety	Revelations	Participants	Number
Somatic Anxiety	Minds going blank	Alice, Carol, Emily, Fiona	4
	Fast heartbeat	Bella, Carol	2
	Increase of body temperatures	Alice	1
	Decrease of body temperatures	Fiona	1
	Confused feelings	Bella, Doris, Emily	2
Avoidance Behavior	Reluctance to write	Emily	1
	Rushing to finish	Emily	1
	No proofreading	Emily	1
Cognitive Anxiety	Fear of low scores	Alice	1
	Fear of other people refusing to read her essays	Bella	1
	Fear of negative comments	Carol, Doris	2
	Fear of low GPA	Fiona	1

#### Somatic anxiety in L2 writing process

During the L2 writing process, Somatic Anxiety was reflected in several symptoms, including minds going blank, confused feelings, change in body temperatures, and pounding heart.

The minds of Alice, Carol, Emily, and Fiona went blank several times during the think-aloud session. When Alice’s mind went blank, she gazed for a long time, or complained:

I am nervous, and I don’t know how to write the essay. Now I have no idea at all. I keep thinking but still have no idea. (Alice)

Alice later confirmed that her blank mind made her only dare to murmur instead of speaking out her thoughts loudly. Alice also declared that it was because of her blank mind that she kept repeating the same words and performed unconscious behavior like sniffing her nose. So did Carol, who confirmed in the interview that her anxiety prevented her from coming up with sufficient ideas, which made her feel that there was nothing in her mind while she was writing. Emily repeated:

I feel so panic, and I don’t know how to write or what I am writing. What can I write? I don’t know. (Emily)

Fiona confirmed that her blank mind led to many other symptoms observed during the think-aloud, including coughing, smacking lips, sniffing nose, biting nails, scratching head, and tapping on the keyboard.

Another symptom was becoming confused, experienced by Bella, Doris, and Emily. When Bella felt confused, she uttered illogical and fragmented sentences, and once came up with sentences that were long, redundant, and grammatically incorrect. Emily also felt confused several times, and she finally composed an essay that contained only one paragraph, which was not clear or orderly. She was aware of this situation herself, and complained that she felt anxious. Before starting her writing, Doris uttered:

I’m a little bit nervous and my mind is a little bit confusing now. Well, let me make an outline first. (Doris)

After finishing her outline, Doris claimed that her mind was clear and began her formal writing.

Somatic Anxiety also affected students’ body temperatures in their second language writing process, leading to either an increase or a decrease of body temperatures. Alice sweated while she was writing, and she later confirmed that she always felt burning when she became nervous or anxious, especially while taking a writing test. By comparison, Fiona once shivered and said that she felt cold during the think-aloud session. Fiona confirmed in the interview:

I often feel cold and have a headache when I try hard to come up with ideas while completing assignments of writing courses. (Fiona)

Participants also experienced fast heartbeat. Alice confirmed in the interview that when she felt extremely nervous, her heart would beat fast. Carol said that she worried about having written too much or too little depending on the topic. Under those circumstances, she would check the number of words continuously, and then became panic, and her heart would beat fast.

When participants were given the definition and description of Somatic Anxiety in the interviews, all the participants admitted that they did suffer from Somatic Anxiety during writing. However, they also claimed that their Somatic Anxiety was not severe, especially compared with Cognitive Anxiety.

As such, the revelations of Somatic Anxiety were not surprising. All the symptoms were quite common among students with different cultural backgrounds. Aichhorn and Puck (2017) listed some universal physical symptoms of anxiety, such as “perspiration including sweaty palms and feet, jittery hands and feet, a dry mouth, and an increased pulse” (p. 751). These universal physical symptoms also encompass many symptoms of Chinese EFL learners found in the present study. Thus, the Somatic Anxiety experienced by Chinese EFL learners did not seem to be culturally unique.

#### Avoidance behavior in L2 writing process

During the think-aloud sessions, most of the participants did not experience much Avoidance Behavior, as they did not show any contempt for second language writing, and some of them actually enjoyed the writing process. Through the think-aloud session, although Alice seemed nervous sometimes, she also laughed several times and showed enjoyment. Bella also said that she did not feel annoyed frequently during the writing process. In spite of her occasional stress, Carol still considered this writing process as interesting, and exclaimed:

How interesting the process is! I really enjoy doing this! (Carol).

Although the whole think-aloud session of Doris and Fiona lasted for 85 and 59 min respectively, they often smiled, remained patient, and regarded this writing process as interesting and pleasing.

The only participant who did not enjoy her writing process was Emily. Through the think-aloud session, she seemed to be reluctant to write, and wanted to finish the essay as soon as possible. Although there was no time limit, Emily rushed to complete her essay in 31 min, with only 202 words. She did not bother to divide her essay into different paragraphs, nor was she willing to proofread her essay or add more details and examples. Her behavior during the think-aloud session posed a sharp contrast to her performance during the interview when she was quite talkative and provided long answers to most of the questions. This contrast suggests that she was not reluctant to communicate with people or participate in the present study, but only not willing to write English essays.

This contrast could be traced to participants’ writing attitudes and habits in their daily life, which were referenced by them in the interview. Emily, the only participant that did not enjoy writing during the think-aloud session, later revealed in the interview that she felt reluctant to write English compositions in her daily life. She habitually answered online essay questions with Chinese. On the contrary, other participants reported that they were willing to compose English essays. For instance, Bella practiced English writing apart from tests and assignments. Doris expressed her willingness to practice English writing, especially in the field of literature. Fiona watched American TV theories or original works of literature, in order to enhance her ability to think in English, thereby sharpening English writing skills.

When participants were given the definition and description of Avoidance Behavior in the interviews, Only Emily claimed that she did suffer from Avoidance Behavior during writing. The other five participants all claimed that they hardly experienced Avoidance Behavior.

The findings regarding Chinese EFL learners’ experience of Avoidance Behavior in the present study are in line with those of previous research in some aspects. As has been mentioned, according to some of the previous studies, EFL learners outside China usually experienced Avoidance Behavior the most (Xiao and Wong, 2014; Salem, 2022), while Chinese EFL learners did not. It will be overly arbitrary to regard Chinese EFL learners’ experience of Avoidance Behavior as different from all those EFL learners outside China. Nevertheless, this finding implies the possibility that Chinese EFL learners are culturally unique in certain aspects of Avoidance Behavior to some extent.

#### Cognitive anxiety in L2 writing process

Most of the participants experienced Cognitive Anxiety, manifested in various ways, including fear of low scores or low GPA, of other people’s negative comments, and of other people’ refusal to reading one’s essays.

For Alice, Cognitive Anxiety was reflected in her fear that she would be negatively evaluated or would receive low scores. In the think-aloud session, she kept criticizing the quality of her essay, and said that this essay would receive a low score. While she was writing, she kept negating herself, such as deleting her phrases and rewriting many sentences. She nearly rewrote the whole paragraph of her third argument. Alice also expressed her regret of having taken this stand. She declared that she would have been able to write better if she had taken the opposite stand. In this way, Alice seemed concerned about other people’s opinions. In the interview, Alice admitted that she behaved similarly when she wrote English essays in her daily life, and that she really cared about scores and other people’s comments. When she was writing, the thought that her essay might be criticized in class worried her. Alice also said that when she was writing, she felt panic and uneasy resulting from the thought that her essay would be scored.

When asked about whether she cared about writing scores, Fiona smiled, and said:

Well, that depends. I always think about scores when I write papers for compulsory course. It annoys me a lot! I often feel extremely nervous at that time. But the scores of elective course are not so important. I don’t care about that, and I don’t spend much time on the papers for elective courses. (Fiona)

Fiona admitted that she would consider the matter of scores when she composed papers for compulsory courses, and she felt extremely nervous in this process, as the scores of these compulsory courses would be counted into GPA. By comparison, when it came to elective courses, she would not spend so much time on her writing, nor would she feel so anxious, as the scores of these elective courses would not be counted into GPA. What she cared about was not the score itself, but her GPA.

Carol and Doris said that they worried about negative comments in their writing process. Carol struggled to improve her essay by seeking different ways to express the same concept. She said that she wanted to make her expressions more diversified, and that repeating the same word would bore the readers. Carol also tried to substitute complicated and difficult words for those simple words in order to make her essay more formal and professional. She once uttered:

My words are too simplistic. I have been using these simplistic words. I should definitely find some complicated expressions. Fine, I’m unable to find a better word. But I do want to find a better word. (Carol)

In the think-aloud session, Doris was extremely careful about her diction, grammar, and logic, and repeated her fear that her essay would be criticized by others. She checked her previous sentences frequently during her writing, and rewrote some of her phrases in this process.

For Bella, Cognitive Anxiety mainly consisted in the fear of other people refusing to read her essays. She said she was afraid that she would make simple spelling mistakes that might result in other people’s reluctance to read her essay. In the think-aloud session, Bella repeated some of her words several times, or read out her previous sentences again before she continued her writing. She spelled out many of the words, and tried to make her essay grammatically perfect. She also said that her sentences might be grammatically incorrect regarding the tense, which would make other people not willing to read her article. Bella said in the interview that during her writing process, when she thought that her essay would be presented in class, she felt pleased, and spared no effort to polish her introductory paragraph, so that her classmates would continue reading her essay rather than stopped reading after seeing the introduction.

By comparison with the other five participants, Emily did not experience much Cognitive Anxiety. Although she was not absolutely confident about her essay, she did not worry about other people’s negative evaluation. She said in the interview that she did not think about the issue of scores when she was writing. Also, it did not matter whether the essay would be scored or not.

When participants were given the definition and description of Cognitive Anxiety in the interviews, all the participants except Emily admitted that they did suffer a lot from Cognitive Anxiety during writing. They also considered Cognitive Anxiety to be the most severe dimension of anxiety experienced by them during the think-aloud sessions.

The findings regarding Chinese EFL learners’ experience of Cognitive Anxiety in the present study are also in line with those of previous research in some aspects. As has been mentioned, according to some of the previous studies, EFL learners in China usually experienced Cognitive Anxiety the most (Chiang, 2012; Sun and Fan, 2022; Zhang and Zhang, 2022), while those outside China did not.

Moreover, Chinese EFL learners’ fear for other people’s refusal to read their essays as well as their fear of low GPA were hardly seen in the samples of EFL learners outside China regarding anxiety. These might shed light on some unique features underlying Chinese EFL learners’ Cognitive Anxiety to a certain degree.

In addition, fear for negative evaluations, one of the revelations of Cognitive Anxiety occurred at a young age for Chinese EFL learners, even in high school. Research showed that Chinese senior high school students experienced anxiety caused by fear of negative evaluation, which is a predictor of self-rated foreign language proficiency (Dong et al., 2022).

Again, it will be overly arbitrary to regard Chinese EFL learners’ experience of Cognitive Anxiety as different from all those EFL learners outside China. Nevertheless, this finding implies the possibility that Chinese EFL learners are culturally unique in certain aspects of Cognitive Anxiety to some extent.

### Variables related to L2 writing anxiety

Participants’ behavior and comments indicated three types of variables associated with L2 writing anxiety. Apart from the learner-internal and teacher-related variables recognized by Jiang and Dewaele (2019), there were also human-external variables, summarized in Table 2.

**Table 2:** Variables related to SLWA.

Category	Variables	Participants	Number
Learner-internal variables	Low self-esteem	Alice, Carol, Doris	3
	Negative attitudes to English	Emily	1
	Perfectionism	Fiona	1
Teacher-related variables	Teacher’s considerateness	Alice, Emily, Fiona	3
	Teacher’s strictness	Carol, Doris	2
Human-external variables	Time constraint	Alice, Bella, Carol	3
	Difficulty of the topic	Carol, Emily	2

#### Learner-internal variables

Learner-internal variables included low self-esteem, perfectionism, and negative attitudes towards English. Low self-esteem was a prominent variable. In the writing process, Alice frequently complained that her essay was poor and messy, that her words were too simple, and that her arguments were not logical and convincing enough. She expressed her fear that her essay would be negatively evaluated because of these drawbacks, and she did not know how to improve her essay. Doris also expressed her fear that her ideas would be overheard by others, who might laugh at her opinions, which were not mature enough, as she considered herself as not qualified enough to write good English essays. Carol thought she had poor grammatical knowledge and repeatedly complained:

What could I do without the word-spelling check function! I have to rely on the software’s automatic word-spelling check function! I feel so bad about my grammar in writing! (Carol)

Perfectionism was another variable. In the writing process, Fiona kept checking her words and expressions. She said that she could not help focusing on these details even though she knew that she should complete her essay before doing the editing work. In the interview, she confirmed that this was a major problem in her L2 writing, which annoyed her in her English writing practice. She stated:

I am a real perfectionist in English writing. I do care a lot about trivial things. I cannot help paying attention to them. (Fiona)

Thus, Fiona always kept polishing her previous sentences and avoiding digression in her writing practices, and she was extremely careful about diction and grammar. Moreover, when Fiona tried to find evidence and examples to support her argument, she tried to come up with interesting and innovative ideas that could make her essay creative. When she could not come up with a “perfect example” at once, she sweated and mumbled that her mind went blank. Fiona admitted that when she was writing English compositions in her daily life, the thought that her essay would be presented in class made her extremely nervous. She would elongate the length of her writing time, criticize herself, and push herself to write perfect articles. This echoes Chiang’s (2012) finding that perfectionism was significantly related to overall writing anxiety, as well as Somatic Anxiety and Cognitive Anxiety.

Besides, negative attitudes to English could be regarded as a variable, which was also recognized by Jiang and Dewaele (2019). Emily, the only participant who showed high levels of Avoidance Behavior during the writing process, habitually used Chinese when answering online essay questions for her courses. She said in the interview that she sometimes considered English as a hindrance to convey her opinions. Alice, the other participant habitually using Chinese when answering online essay questions, claimed that she did not think English would make it difficult for her to convey messages. The reason for choosing Chinese was only that she could convey messages more clearly and easily in Chinese. In other words, she did not “dislike” English, but only “preferred” Chinese. That might explain why she did not show severe symptoms of Avoidance Behavior like Emily. The other four participants who habitually used English when answering online essay questions all claimed that since the questions or instructions were given in English, they would also prefer to write in English to make their texts more logical and authentic.

Learner-internal variables, namely low self-esteem, perfectionism, and negative attitudes do not seem unique themselves. Young (1991) and Dewaele (2017) regarded self-esteem and perfectionism as universal variables related to anxiety. Dovchin (2020, 2021) recognized self-esteem as related to Mongolian EFL learners’ anxiety. Actually, the way cultural factors shape the uniqueness of Chinese EFL learners regarding these variables might be reflected in the degree or extent. Perfectionism occurred at a young age for Chinese EFL learners, even when they are in high school (Chiang, 2012). As for the negative attitudes to English, this phenomenon only occurred in the performance of one of six participants, suggesting that this might not be a severe issue for Chinese EFL learners.

#### Teacher-related variables

Teacher-related variables concerned the habits or personality of teachers, such as strictness and considerateness. Carol mentioned that one of her writing teachers (who was Chinese) once presented her essay in class and pointed out the problems. She felt embarrassed as her name was revealed. Since then, when she thought about this in the process of second language writing, she felt extremely uncomfortable. Doris also said:

When I write my essays, I often imagine that my essays will be presented in class. I am very scared by this thought, and I worry a lot about it. I don’t want other people to say something bad about my essays. If my writing teacher does not reveal my name, then that’s fine. I can pretend that this assignment is not mine. (Doris)

Carol and Doris also mentioned that when they were in middle school, their English teachers (who were all Chinese) always revealed their names when openly criticizing their essays, which made them really anxious.

On the contrary, Alice mentioned that when her writing teacher (who was a westerner) presented their essays in class and pointed out their disadvantages and problems, he never revealed the names of the authors, and did not count this into their scores, so she did not become anxious. Emily expressed similar ideas, and said she considered this anonymous presentation as beneficial, which made it possible for students to review each other’s works. Fiona mentioned that her essay was once presented by the teacher of an English course, along with many essays composed by other students. As their essays were presented informally in a social network chatting group, she did not feel uneasy, but enjoyed reading those essays. In her opinion, the presentation of essays in this way was beneficial, as it could help students communicate with their classmates and learn from each other.

Moreover, teachers’ restrictness or considerateness could be reflected in giving feedback. Carol and Doris mentioned in the interview that their Chinese teachers of English usually criticized the flaws of their essays in the feedback. They did regard these suggestions and comments as helpful, but they admitted that such criticism ignited their anxiety tremendously. Different from Iranian teachers of English who gave feedback, feed up (comments on goals and students’ success in achieving goals, e.g., “You have done your task successfully. This enumeration paragraph is good.”) and feed forward (comments on the next step in learning during the semester, e.g., “In the next session, we will learn how to write supporting sentences.”;) (Zarrinabadi and Rezazadeh, 2020), Chinese teachers of English usually give feedback, rather than the more encouraging feed up or feed forward. Since feed forward was suggested to significantly lower students’ writing anxiety (Zarrinabadi and Rezazadeh, 2020), writing anxiety of Iranian EFL learners could be lowered by their teachers’ habits of giving feedback, while writing anxiety of Chinese EFL learners could not be lowered in the similar way.

In this sense, teacher-related variables, which involved the habits or personality of teachers, mirrored the differences between some of the Chinese teachers and western teachers to some extent. Again, these differences should not be regarded as absolute, since all countries can have strict and considerate teachers disregarding their cultural background. The cultural uniqueness of Chinese EFL learners in this regard should still be a matter of degree.

#### Human-external variables

Human-external variables included the existence of time constraint, and difficulty of the topic. Alice, Bella, and Carol wrote under the time constraint during the think-aloud session. Alice seemed stressful during her writing, until she finished the first draft and began to revise. She later confirmed that at the beginning, she felt extremely worried about running out of time, but when she realized that there were still about 10 min left after finishing with 406 words, she was not so anxious.

Bella began to feel uneasy when she started to write the third body paragraph. She began to make shorter and faster sounds, or gazed upwards and played with her fingers. She later confirmed:

My mind began to go blank as time was running out. I went panic and suddenly couldn’t find anything to write. (Bella)

Later, Bella retrieved her calm and fluent voice after she checked the number of words and found that she had written more than 300 words. She said:

I often check the number of words when I feel panic in writing. If I find that I am about to reach the required number, I can feel relaxed. (Bella)

Bella remained calm in the rest of the time, and finally finished her essay in only 20 min, with 410 words.

Carol said that she was always thinking about the matter of time in her writing process, which made her nervous, until she became immersed into her work. She finally finished her essay with 300 words in 29 min. She said:

If I had had more time, I would have become less panic and would have written more smoothly with more words. (Carol)

Carol said that if she had had abundant time to compose her essay, she would not have experienced these symptoms. When she wrote essays for some writing courses, for example, she usually had a couple of days, so that she seldom felt nervous while completing those articles.

By comparison, there was no time limit in the writing process of Doris, Emily, and Fiona. Doris did not rush to write her essay immediately after reading the instruction, but kept drafting an outline in mind for 10 min, always thinking in a careful, logic and calm way. She also thought about possible details and examples that could be used in her essay. She carefully considered what the opponents might rebut, and pondered upon how to respond to their rebuttal. Through the think-aloud session, the only sign of Somatic Anxiety occurring in Doris’ performance was the confused feelings displayed before she made her outline. During her later writing process, she did not sweat, shiver, or show other signs of physiological symptoms of anxiety. She uttered most of her thoughts logically and orderly, and always focused on details stated in the instruction. Doris, Emily, and Fiona did not bother to check the time or the number of words, and finished their essays in 85 min with 359 words, in 31 min with 202 words, and in 58 min with 315 words, respectively.

Another variable was the difficulty of the topic. Carol complained several times:

I do not know what to write, as this topic is too difficult! How can I write this! (Carol).

Carol mentioned that she considered this topic (i.e., “whether daily homework is necessary for students”) to be difficult, because she was used to doing homework every day in the past years. It never occurred to her that this could be debated. She also mentioned that her writing teachers sometimes assigned difficult topics which could be very unfamiliar to many Chinese students, such as policies exclusive to western countries. She hardly thought about these issues and could write nothing about them. These topics made her really anxious.

Emily repeatedly emphasized that she could only write down her random thoughts in an illogical and unconvincing way due to the difficulty of the topic. Emily revealed in the interview that she also felt reluctant to write English compositions in her daily life, mainly because she felt the assigned topics difficult and uninteresting. She also confirmed in the interview that she often encountered problems of this kind, especially when the topic involved issues in other countries, to which she was not familiar and lacked sufficient resources.

Again, human-external variables themselves which concerned the time constraint and the difficulty of topics were not culturally unique for Chinese EFL learners. The possible uniqueness might lie more in what counts as difficult. For Chinese EFL learners like Carol and Emily, the topics involving western-exclusive phenomena could be very difficult for them, increasing anxiety.

## Discussion

Findings of the present study are in line with the third trend mentioned in “Trends of research on anxiety,” concerning whether anxiety is internally decided or socially constructed (MacIntyre, 2017). In the present study, three types of variables related to anxiety were identified, including learner-internal, teacher-related, and human-external variables. Participants’ L2 writing anxiety kept interacting with these variables in a dynamic way, especially time constraint. Alice, Bella, and Coral wrote under time constraint, and their anxiety fluctuated. To be more exact, they felt more anxious when they were not sure whether their time was sufficient, and less anxious when they had already reached the required length and thought time was under their control. This would appear to lend support to the dynamic approach to anxiety referred to in MacIntyre’s (2017) review. This might not be culturally unique to Chinese EFL learners per se, but the exact variables in the dynamic construction of anxiety might be affected by cultural factors to some extent.

For Chinese EFL learners, the revelations of the three dimensions of SLWA as well as the variables related to SLWA show differences in some aspects from those EFL learners outside China, though the differences should not be regarded as fundamental. Therefore, the uniqueness of Chinese EFL learners’ SLWA might be a matter of degree, and is reflected in certain aspects of different dimensions of SLWA, as well as some variables related to SLWA, mirroring the influence of cultural factors, summarized in Table 3.

**Table 3:** Cultural influences on SLWA.

Dimensions of culture	Cultural influences	Chinese learners’ experiences
Ethnic culture	The respectable position of Chinese teachers, leading to the distance between teachers and students in China	Cognitive Anxiety raised by teachers’ presentation of their essays without concealing their names; teachers’ strictness, a teacher-related variable related to anxiety (Carol, Doris)
	Chinese people defining the achievement of young people in academic terms	fear of low scores or low GPA, increasing Cognitive Anxiety (Alice, Fiona)
	Chinese virtue of modesty	Low self-esteem, a learner-internal variable related to anxiety (Alice, Carol, Doris)
	Chinese virtue of diligence	experiencing much Cognitive Anxiety and little Avoidance Behavior (all but Emily)
Local culture	Chinese students feeling difficult when lecturers choose examples outside China as an illustration	anxiety resulting from difficulty of the topic, a human-external variable related to anxiety (Carol, Emily)
	Chinese local training and education atmosphere attending more to exams, rather than to extra-curricular activities	experiencing much Cognitive Anxiety and little Avoidance Behavior (all but Emily)
Academic culture	Scarcity of writing centers so that Chinese students having to rely on academic peer help (also Confucian values and collectivist approach)	cherishing the in-class peer review and worrying that other students would refuse to read one’s essays, increasing Cognitive Anxiety (Bella)
	Regarding flawless performance as the academic goal	the cultivation of perfectionism, a learner-internal variable related to anxiety (Fiona)
Disciplinary culture	Exam-oriented culture in Chinese educational context, attaching greater important to written English than oral English, particularly obvious to English majors	English majors trained to write different kinds of English essays, paying attention to the cultivation of English writing habits, reducing Avoidance Behavior (Bella, Fiona)

Ethnic cultural influences consisted in the respectable position of the teacher, the strong achievement motivation, and the Chinese virtue of modesty and diligence. Strictness and considerateness of the teacher were indicated as variables related to anxiety. Carol’s and Doris’ experiences showed that their Cognitive Anxiety was raised by teachers’ presentation of their essays without concealing their names. Since the Confucian heritage emphasizes the respect for teachers (Bond, 1986, 1991), Carol and Doris did not negotiate with their teachers to remove the names of the authors before presenting the essays. The respectable position of teachers also involves teachers’ ways of giving feedback, which are often strict, increasing Carol’s and Doris’ Cognitive Anxiety. The respectable position of teachers also leads to the teacher-student distance. It is thus difficult for teachers to “light the students’ fire” in class (Dewaele et al., 2018, p. 694), in order to generate students’ positive emotions and alleviate negative emotions including anxiety (Dewaele et al., 2018, 2019).

In addition, strong achievement motivation is a significant feature of Chinese ethnic culture (Bond, 1986, 1991), since Chinese people often define the achievement of young people in academic terms (Flowerdew and Miller, 1995). Essau et al. (2008) pointed out that “being raised in collectivistic culture which places high value on education and filial piety, Chinese adolescents are pressured to achieve academically not to disappoint their significant others and to maintain their social identity” (p. 803). These could partly explain why several participants experienced severe Cognitive Anxiety, regarding fear of low writing scores or for low GPA.

Chinese virtue of modesty, another feature of Chinese ethnic culture, might result in low self-esteem occurring in the cases of Alice, Carol, and Doris, when some students go too far. Because of the virtue of modesty, students might also find it hard to use positive self-talk, a strategy to alleviate anxiety (Toyama and Yamazaki, 2021).

Ethnic culture also concerns Chinese virtue of diligence. Chinese people believe in the idea of achieving success through hard work. Students who want to pursue success should be more devoted to hard work. In the present study, the Cognitive Anxiety experienced by Alice, Bella, Carol, Doris, and Fiona reflected their goal for high evaluations, scores, or GPA. Consequently, they might be more willing to work harder, so that they did not display many revelations of Avoidance Behavior. By comparison, Emily might attach greater importance to life than work, so that she did not care much about evaluations, scores, or GPA, and displayed little revelations of Cognitive Anxiety. As a result, she might find it not interesting to write English essays, and displayed many revelations of Avoidance Behavior.

Local cultural influences consisted in the unfamiliarity with other countries. It is common for Chinese students to feel difficult when lecturers choose examples outside China as an illustration (Flowerdew and Miller, 1995). Similarly, many L2 writing topics assigned in Chinese class involve issues in the USA or the UK, increasing the difficulty of the topics, since Chinese EFL learners are generally less familiar with these countries compared with their counterparts in the western countries. Chinese students have fewer chances of direct interaction with members of the target culture, and face steeper cultural barriers, compared with their international counterparts (MacIntyre et al., 2019). Carol and Emily both mentioned that the topics concerning issues in other countries would appear difficult to them, due to their unfamiliarity and lack of sufficient resources. Since the difficulty of the topic was identified as a variable related to anxiety in this study, this would increase their anxiety.

Local culture in China also encompasses the phenomenon that Chinese local training and education atmosphere attends more to exams, rather than to extra-curricular activities. College Entrance Examination in China focuses on scores rather than on extra-curricular activities as in some foreign countries. Thus, the local training in China is usually more academic and serious, while local training in some western countries might take the form of some entertaining activities, such as drama, a good way to alleviate anxiety (Toyama and Yamazaki, 2021). Because of this kind of local setting, Chinese EFL learners care about their scores, experiencing much Cognitive Anxiety, but they have become accustomed to hard work, thereby experiencing less Avoidance Behavior. In Xiao and Wong’s (2014) study, participants were Chinese heritage language learners, so that they shared the similar ethnical cultural identity with Chinese EFL leaners. As these participants were studying in American universities, however, the local culture should be different from that in China. As a result, these learners actually experienced Avoidance Behavior the most rather than Cognitive Anxiety, a pattern similar to EFL learners outside China.

Academic cultural influences consisted in the practice of academic peer help. In some other countries, there are writing centers which provide tutoring service for students. In China, on the contrary, this kind of writing centers could be rather rare. Instead, students tend to rely on peer help, also mirroring the Confucian values (Flowerdew and Miller, 1995) and collectivist approach (e.g., Matsumoto et al., 1988; Stipek, 1998; Frenzel et al., 2007; Essau et al., 2008). Thus, participants cherished the in-class peer review. Jin and Dewaele’s (2018) work on Chinese EFL students further indicated that perceived peer support was related to reduced anxiety to a greater extent than support from teachers, and this further suggested Chinese EFL learners’ favor for academic peer support. Toyama and Yamazaki’s (2021) review regarded Student–Student interactions as indispensable to reduction of anxiety. These could explain why Bella worried that her essay would be so uninteresting that other people would refuse to read her essays, depriving her of an opportunity to acquire detailed advice from others apart from teachers. Her fear of other people refusing to read her essays was an aspect of Cognitive Anxiety, as has been mentioned.

Academic culture in China pursues flawless behavior. This is reflected in the scoring mechanism of exams in China and those in western countries. Generally, every mistake in exams in China will lead to the deduction of points. By comparison, some exams outside China have the “fault tolerance rate,” which means that a test-taker could still obtain full marks despite a few mistakes. The pursuit for flawless behavior may result in perfectionism, reflected in Fiona’s performance, as well as in the performance of some Chinese students at a young age (Chiang, 2012).

Disciplinary cultural influences consisted in abundant writing practices. Chinese educational context possesses an exam-oriented culture, attaching greater important to written English than oral English (Jiang and Dewaele, 2019; MacIntyre et al., 2019), and this is particularly obvious to English majors (or at least to the participants of the present study). Thus, Chinese EFL learners have abundant opportunities to practice English writing, especially for English majors, including the six participants. They had been trained to write different kinds of English essays, and they paid attention to the cultivation of English writing habits. As has been mentioned, Bella wrote English essays not only for homework, but also for her personal practices. Fiona practiced English writing skills by learning from original works of literature and TV series. These are actually representative practice or routines of English majors in China. Consequently, most of them have become used to English writing, which could explain the low degrees of Avoidance Behavior observed in the think-aloud session.

Again, it will be overly arbitrary to claim that the abovementioned cultural factors never occur in other countries. What the discussion above truly suggests is that these cultural factors tend to be more prominent in China than in many other countries, so that they could help shape Chinese EFL learners’ uniqueness in some aspects of SLWA, and such uniqueness is also a matter of degree.

## Conclusion

The present study has investigated Chinese EFL learners’ three dimensions of second language writing anxiety and the related variables, exploring the cultural uniqueness. Participants exhibited obvious Cognitive Anxiety, but relatively subtle Avoidance Behavior.

Three types of variables dynamically interacted with L2 writing anxiety. Learner-internal variables encompassed low self-esteem, perfectionism, and negative attitudes towards English. Teacher-related variables encompassed the habits or personality of teachers, such as strictness and considerateness. Human-external variables encompassed time constraint, and difficulty of the topic. These findings indicate the uniqueness of Chinese EFL learners’ SLWA in certain aspects of different dimensions of anxiety, or some variables related to SLWA, mirroring the influences of cultural factors, though the differences are not fundamental, and are more of a matter of degree. Firstly, participants respected and were distant from their teachers, strongly worried about low scores and low GPA, and cherished the Chinese virtue of modesty and diligence, increasing Cognitive Anxiety but not Avoidance Behavior, mirroring ethnic cultural influences. Secondly, writing topics concerning issues in western countries appeared difficult to participants since they were unfamiliar with other countries, and students have been familiar to Chinese local training that focuses on exams instead of extra-curricular activities, mirroring local cultural influences. Thirdly, students relied on the valuable in-class peer review, and pursued flawless performance, influenced by academic culture. Fourthly, most of the participants showed slight Avoidance Behavior, benefiting from their writing habits fostered by abundant writing training as English majors in China, and this reflected disciplinary cultural influences.

As a qualitative study, the present research has several limitations. Firstly, due to the small sample size, the factors recognized in the present study were relatively limited. Quantitative research could be combined to display more factors. Secondly, participants all majored in English, so that results of the present study could only showcase the L2 writing anxiety experienced by this single major, while the conditions of non-English majors were not explored. Future research could investigate Chinese EFL learners from other majors to further examine the uniqueness and cultural influences.

Despite these limitations, the present study could provide pedagogical implications. For teachers of Chinese students, they could first be friendlier and encourage students to express their feelings and needs. When presenting students’ essays, teachers could conceal their names, and remember to mention the merits, instead of only pointing out the drawbacks. Second, teachers could modify the scoring mode by reducing the weight of writing assignments and tests, and raising the weight of writing attitudes and effort, such as by taking into consideration their frequency of on-line discussion participation. Third, teachers could choose more local and interesting writing topics for students, and provide them with more background information if the topics involve issues in other countries. Fourth, teachers could provide more opportunities of peer review, and encourage students to read each other’s work, no matter it is well-written or poorly-written. Teachers of students in other countries, on the other hand, may also acquire implications from the present study, learning from positive aspects, such as the fostering of writing habits to reduce Avoidance Behavior, and avoiding negative factors, such as the preoccupation of scores and GPA which could increase Cognitive Anxiety. In this way, they can also improve their teaching and alleviate students’ anxiety.

## Data availability statement

The raw data supporting the conclusions of this article will be made available by the authors, without undue reservation.

## Ethics statement

Ethical review and approval was not required for the study on human participants in accordance with the local legislation and institutional requirements. The patients/participants provided their written informed consent to participate in this study.

## Author contributions

YY conceived the idea, analyzed the data, and drafted the manuscript. DZ provided critical feedback and helped to shape the research, as well as reviewed and revised the manuscript. All authors contributed to the article and approved the submitted version.

## Conflict of interest

The authors declare that the research was conducted in the absence of any commercial or financial relationships that could be construed as a potential conflict of interest.

## Publisher’s note

All claims expressed in this article are solely those of the authors and do not necessarily represent those of their affiliated organizations, or those of the publisher, the editors and the reviewers. Any product that may be evaluated in this article, or claim that may be made by its manufacturer, is not guaranteed or endorsed by the publisher.
